# Synergies of affordances and place-based relationality in Forest School practice: implications for socio-emotional well-being

**DOI:** 10.3389/fpsyg.2024.1352374

**Published:** 2024-05-10

**Authors:** Vinathe Sharma-Brymer, Eric Brymer, Royce Willis, Matthew Leach

**Affiliations:** ^1^School of Law and Society, University of the Sunshine Coast, Maroochydore, QLD, Australia; ^2^Faculty of Health, Southern Cross University, Lismore, NSW, Australia

**Keywords:** Forest School, nature-based activities, affordances, Indigenous place-based relationality, socio-emotional well-being

## Abstract

Research shows that the human-nature relationship positively impacts human well-being. Forest School (FS) practice offers young children a structured program of nature connection through activities, aiming to enhance their self-esteem and social skills. FS is now adapted in countries such as Australia, Canada and New Zealand where a unique cultural interface occurs between European settlers and Indigenous peoples. Responding to socio-cultural diversities, geographical contexts, and the traditional ecological knowledges, FS needs to go beyond play pedagogy and incorporate theoretical perspectives that promote human-nature relationship in local context-specific environments. We argue that the synergies between Western perspectives on affordances perceived in person-environment relationship and Indigenous place-based relationality perspective provide a more suitable approach for developing reciprocal relationships between FS participants and land/place/nature. We propose that the synergies between affordances perceived in FS and place-based relationality cultivated in participants will enhance social and emotional well-being. We call for specific research investigating such synergies supporting participant well-being. Future research on FS practice should be directed toward initiating and exploring co-designed studies by Indigenous and non-Indigenous researchers incorporating methodologies that study participant experience as well as evaluating the impact of FS programs embedding affordances and place-based relationality perspectives.

## Introduction

In recent years, research has shown that enhancing the human-nature relationship positively impacts the health and well-being of people and planet. This paper argues that Forest School (FS) practice, a child-centered program of nature-connection that originated in Scandinavia and developed in the United Kingdom (UK), as is now offered in many countries, could be ideal to foster both planetary and people health and well-being because it enhances human-nature relationships in early childhood. However, to reach its potential when it is adapted in different socio-cultural and geographical contexts reflecting diverse traditional ecological knowledges (TEK), FS needs to be underpinned by theoretical perspectives that go beyond the Eurocentric play pedagogy. We argue that the synergies between the Western perspectives on affordances and Indigenous place-based relationality provide a solid theoretical underpinning for FS design and implementation. This will guide effective local context-specific practices enhancing participant well-being outcomes. This is especially important as FS expands from its original Scandinavian roots to countries with a significant cultural interface between European settler populations and Indigenous peoples, such as in Australia, Canada and New Zealand. We call for specific research investigating such synergies supporting participant well-being and the FS program impact.

## Forest School practice and benefits

The FS model, formalized in the UK, drew upon the *udeskole* practice of Denmark which encouraged pre-school aged children’s experiential learning in the outdoors (O'Brien, 2009; Knight, 2012). The Scandinavian philosophy of *friluftsliv*, or ‘free air life’, influenced *udeskole* practice encouraging feelings of being free as part of the natural world with an embedded spiritual connection (Gelter, 2000, 2010; Bentsen et al., 2018). FS is designed to nurture children’s holistic development by fostering a meaningful connection with self, activity and the natural environment. In the UK, the FS ethos draws from play pedagogy and the social constructivist paradigm (Knight, 2018), promoting child-centered experiential learning as a means to enhance self-esteem. It emphasizes young children’s engagement with nature, cultivates peer interactions and social skills, and supports a sense of belonging. This approach positions FS as an effective avenue for the nurturance of children’s self-esteem in early years.

The FS program is designed predominantly for children to immerse themselves in regular weekly nature-based activities of at least 2 hour duration. Most often, the program takes place in natural environments such as woodlands, parks, urban forests, farms, and community green spaces. These sessions incorporate bushcraft, wood whittling, den building, nature-based games, arts and crafts, fire making, and storytelling (Sharma-Brymer et al., 2018). Children enjoy these activities while creating personal connections and meanings with their surroundings (Harris, 2021). They orient their sensory-perception learning to interpret and respond to various environmental stimuli thereby developing their physical, sensory, perception, and cognitive abilities. For example, tree climbing in FS can support multifaceted development including generating personal meanings alongside physical coordination and self-awareness.

Although FS approach is advocated as child-centered, in practice adults design and deliver the program activities focusing on rigorous risk assessment and school curriculum alignment, which arguably diminishes children’s agency and the child-centric philosophy (Sharma-Brymer et al., 2018). In this sense, FS model could look back at its original roots of Scandinavian *friluftsliv* and *udeskole* practices for embracing a spiritual connection of being part of the natural world.

The FS model is now replicated in diverse geographical regions such as Australia, Canada, and New Zealand with local-specific modifications and adaptations [see MacEachren (2013), Cumming and Nash (2015), Alcock and Ritchie (2018), Boileau and Dabaja (2020), and Speldewinde and Campbell (2023)]. These countries offer a unique cultural interface between European settlers and Indigenous peoples. As such, FS needs to be diversified to take into account local Indigenous place-based relationality frameworks as well as keeping its more Western person-environment relationship roots.

## Relationality in human-nature relationships and implications for Forest School

From a place-based relationality perspective, humans relate to nature and other life forms with an inherent sense of connection and belonging. Relating to the natural environment by developing personal connections and meanings benefits individual and planetary health and well-being (Lawton et al., 2017; Brymer et al., 2021; Jimenez et al., 2021). Research has demonstrated that spending time outdoors positively impacts physical and mental health, and life satisfaction (Biedenweg et al., 2017; Barnes et al., 2019; White et al., 2019; Coventry et al., 2021; McCartan et al., 2023). Natural spaces occurring in wilderness, woodlands, parks, and water bodies provide urban dwellers diverse opportunities for self-awareness and self-reflections (Cooley et al., 2021). Nature-based activities can significantly enhance overall well-being across the lifespan. For example, well-designed outdoor activities can raise self- awareness, self-esteem and belongingness in young people (Roberts et al., 2020), while adults participating in community gardening can gain new learning that positively impacts their self-awareness and social connectedness (Gregis et al., 2021). These examples illustrate the positive impact of human-nature relationships across all domains of well-being, strengthening the relationship to self and place which further encourages belonging.

In contemporary fast-paced, technology-driven life patterns, relating to nature has an invaluable benefit for human beings. Extending on this important point, we draw from Australian Aboriginal scholar Graham’s (2014) conceptualization of place-based relationality underlining the perspective of how human identity is intertwined with the natural world. Graham (2014, p. 18) reflects on the connectedness between self and the land by noting “I am located therefore I am”, stressing human-nature interdependency. Graham (2023) also highlights that the human-nature relationship is underpinned by notions of kinship, caring and belonging. This interconnectedness impacts human identity and well-being. This notion is relevant across various Indigenous collectivist cultures which value the significance of the ties between people, place, culture and community. For example, according to Gee et al. (2014), interconnectedness within the context of Australian Aboriginal and Torres Strait Islander peoples’ cultures involves an interplay between land (Country), culture, family, kinship, community, and spirituality in forming identities, and influencing social and emotional well-being.

The FS model provides a rich scope for practically exemplifying the above concepts in action. The intention of the nature-based activities of FS is to facilitate children’s connection with their environment, while simultaneously encouraging social and emotional connectedness. However, this intention is often overlooked by activities thereby suppressing the deeper person-environment connection. This intention should align with Graham’s (2014) view of ‘being and belonging in a place’, when FS participants relate to the local natural environment allowing for connections to Country/land/place and the life forms [also see Thornton et al. (2021)]. For example, young children participating in the weekly Bush Explorers program in Australia (Bunyaville Environmental Education Centre, n.d.) learn about local fish and frogs whilst exploring a local creek. This experience could be extended further by introducing relevant Aboriginal Dreamtime Stories to cultivate cultural curiosity in children about Indigenous knowledges and perspectives related to Country and the interconnections between its life forms. This experience potentially grounds their sense of self in the place.

Developing such relationships can also imply their responses to affordances present within the environment. In this light, we explore individual capacity for perceiving opportunities in the environment emphasizing the person-environment relationship in experiential learning.

## An affordance perspective on Forest School

The FS ethos emphasizes child-centered engagement with activities and environment, encouraging free play, and nature explorations. Guided by adults, children are encouraged to take risks in a safe yet challenging environment. This approach enables children to learn from myriad opportunities present in the natural world. The dynamic and sensory-rich environment offers diverse experiences, including the changing seasons and varied natural stimuli (sounds, smells, and sights), integral to experiential learning. These experiences expose children to the properties of the environment, cultivating specific knowledges that assist in the development of self-awareness, leading to deeper interactions and facilitating a sense of attuning to information in the environment. At an individual level and also in small groups, children come to recognize their relationship with the place and its features as well as with peers. For example, they remember where their favorite tree is across seasons despite seasonal effects on the tree, allowing for perceiving different opportunities to interact with that tree as relevant to the season. They remember the ways of peer play associated with that tree allowing for richer reflections on relationships and meanings.

Gibson’s (1979) ecological psychology, particularly his concept of affordances, is highly relevant to the FS approach of experiential learning. Ecological psychology highlights the dynamic relationship between the individual and their environment. Affordances refer to multiple potential opportunities that the person-environment relationship offers; they are invitations for actions. Gibson conceptualized perceived affordances as action-opportunities within the person-environment relationship available to an individual. “The *affordances* of the environment are what it *offers* the animal, what it *provides* or *furnishes*, either for good or ill” (Gibson, 1979, p. 127; original emphasis). As such, the properties of the environment are perceived as possible action opportunities initiating behaviors, behavioral changes or adaptations (Heft, 1988).

Gibson posited that an individual’s understanding and interactions with their environment are transformative, leading to better self-awareness. This process is driven by ongoing interactions with the environment, continually shaping sensory, perceptual, and cognitive abilities. By alerting oneself to environmental properties, one is able to continually ‘read and perceive’ (attuning) information present in the environment. This gradually enhances the person-environment relationship, learning to perceive opportunities with individual agency to act. The relative person-environment relationship strengthens with developing an intimate knowledge of the place and relating to it in myriad ways (Brymer and Davids, 2014). As children in FS interact with their environment, they learn to perceive the rich landscape of affordances and respond adaptively, developing an acute awareness of the relationship between self and their surroundings.

In FS, children’s engagement with natural environments fosters their perceptual and responsive capability. Participating children, over a prolonged period and regular duration, are active agents who learn to attune to information in the environment, see, listen, smell, taste, and feel the place for its richer array of affordances thereby developing the capabilities to act on the environmental properties in newer ways.

A good example to illustrate affordances in FS is ‘mud kitchen’ play activity using a play kitchen set, mud (soil), fallen leaves, fruits and seeds, flowers and other such organic objects from natural surroundings. The children create numerous sub-plays within ‘mud kitchen’ play using imagination and learning to work collaboratively. This play activity enhances peer interactions, social skills, fostering creativity, curiosity and nature exploration with deeper connections. Additionally, it supports calmness and relaxation. The social, physical, emotional and cognitive domains are interactive, reinforcing personal relationship, constraining behaviors that are responsive to perceiving multiple opportunities (Brymer and Davids, 2014). Affordances present in this play context evoke multiple behavioral responses. For example, mud can become soup in the ‘play kitchen’ or be used as paint of different hues for artwork decorating the kitchen. The action capabilities (Woods and Davids, 2021) stemming from cultural experiences, beliefs and attitudes interact with the effectivities of the environment, constraining emerging behaviors. Children learn about flora, fauna, food types that are human-friendly and animal-friendly, and seasonal changes and their effects on human interactions with the environment, all of which also develop a sense of guardianship and reciprocal relationships.

The interactions with affordances in the human-nature relationship also foster the development of a reflective and relational self, aligning with Graham’s (2014) concept of place-based relationality. This kind of interactional ‘knowing’ the environment intertwines with the experience of ‘being’ in it (perceiving affordances) and doing (actions). This holistic engagement with the environment contributes to their cognitive and emotional development and instills a sense of responsibility and connection to the natural world. The following section discusses the alignment between affordances and place-based relationality to enhance FS practice and experience.

## Synergies between affordances and place-based relationality in Forest School

The FS nurtures a unique connection between children and nature, promoting an appreciation of the natural world. This connection is fostered through immersive experiences where children sense and feel, listening to woodland sounds, touching and feeling the textures of trees, plants, leaves, flowers, fruits and seeds on the forest floor, while observing subtle changes in their surroundings. When explored through an integrative lens this immersive exploration fosters a place-based relationality, where children learn to effectively attune to information in the natural environment, thereby developing their capacity to perceive, relate and action helpful affordances. This further develops their sense of self in place.

While reflecting on ‘place’ (landscape within the natural world), Thornton et al. (2021, p. 5) allude to “place attachment, place meaning, and place-responsiveness,” asserting humans are part of and belong to the natural world. Graham (2014) emphasizes coherence in one’s sense of identity and belonging through people-place relationality. When we sense and feel the place coherently, we relate to it with confidence. Our “social, spiritual and cultural life” (Graham, 2014, p. 19) shapes our relationships, influencing such coherence and confidence both in turn influencing our identity. This fosters a sense of security which comes from a deep reciprocal affiliation with the place ‘where we belong’ and custodial care for our place. From a Native American perspective, Kimmerer (2013) also highlights such reciprocity, a foundational understanding in human-nature relationships with respectful interactions in the natural world. Sharing Māori worldviews from New Zealand, Watene (2016) underlines the importance of local-specific TEK in supporting humans’ meaningful relationships with the natural world.

These concepts are significant to the notion of affordances. Attuning to information in the environment and perceiving the rich landscape of affordances available in the person-environment relationship strengthens action possibilities as well as a meaningful and reciprocal relationship with nature. Knowledge about the environment grows from direct experience, that is deeply relational and reciprocal (e.g., looking after the place).

Reflecting on the custodial ethic of care, Thornton et al. (2021, p. 5) state it “emerges out of place and is also a structuring force,” impacting on human well-being. Graham (2013, 2023) emphasizes the harmonious and caring interactions with that force in all its variations. The affordance notion provides some detail to this understanding as attuning to information in the environment and strengthening the capacity to perceive and action affordances also facilitates a relationship whereby protecting affordances for good becomes paramount. The interactive, interdependent and relational relationships connecting self, performance and the environment are embodied and also facilitate a sense of responsibility and stewardship. The intrinsic value of this embodiment is reflected in knowing the place and the place growing within us influencing the formation of our identity and belonging.

In affordance theory, the individual’s relationship with the natural environment is complex, shaping knowing, being and belonging experiences, as well as the relationship with the natural world, oneself, and others. This holistic approach to learning and development informs how best to facilitate experiences that support Graham’s (2023) view of a mutual understanding and connection between the individual and environment: “… the relation between people and land becomes the template for society and social relations” (Graham, 2008, p. 182). Graham also underlines the need for developing a collective spiritual identity through our connection to land/place. Affordance theory suggests collective spiritual identity through enhanced perception and action of affordances for behavior, principles of ethics and values of caring for the place in its holistic sense. FS can support these notions encouraging participants to develop reciprocal responsibility and social relations through deeper connections with self, peers, and the place. These integrated perspectives are crucial for social and emotional well-being.

## Implications of synergies for socio-emotional well-being

The FS offers a vital counterbalance to the challenges of a sedentary lifestyle and digital overexposure, which increasingly affect children’s social and emotional well-being (Roberts et al., 2017). A review by Dabaja (2022) highlighted the positive impact of FS on children in improving social, cooperative, and physical skills. Studies by Cumming and Nash (2015) and Coates and Pimlott-Wilson (2019) document behavioral transformations and enhanced social dynamics in the FS setting. Interviews with FS practitioners (Harris, 2023) reveal shifts in communication skills and interaction styles, benefiting children who struggle with shyness or anxiety. The FS environment thus becomes a powerful set of activities for enhancing cooperation, calmness, confidence, and relationship building.

From a Western viewpoint, affordance theory is pivotal in FS, enabling children to recognize and utilize learning opportunities within the person-environment relationship. Equally important is how affordance theory supports the Indigenous place-based relationality concept in colonial-settler societies. Researchers from Australia, Canada, and New Zealand have underlined the importance of outdoor learning in early childhood education incorporating Indigenous worldviews. FS is popular in Australia where early childhood educators have modified it with ‘bush kindergartens’ (bush/land/place/Country) and ‘nature play’ approaches for local-specific adaptability (for example, Bush Kindy FS, Bush Knowing FS, Bush Explorers FS etc.) [see Speldewinde and Campbell (2023)]. Responding to the importation of Forest School model into New Zealand, Alcock and Ritchie (2018) noted the significant role of Māori knowledges in enriching children’s experience in the outdoors. Similarly, MacEachren (2018) argued for decolonizing early childhood pedagogies in Canada by adopting Indigenised place-based, relational First Nations’ pedagogies. In the Australian context, Christiansen et al. (2018, p. 69) emphasized diverse nature kindergarten sites enriching “localized sets of place-based relationships” and recognizing the local Aboriginal cultural connections to the bush. First Nations educator-led programs such as On Country Learning in Australian context (Lee-Hammond and Jackson-Barrett, 2017) and The Ngahere kindergartens in New Zealand (Kelly et al., 2013) demonstrate the value of place-based relationality where children develop a deep interconnected relationship with the place. These occasional studies demonstrate a dearth in research exploring the incorporation of Indigenous worldviews in FS adaptations.

Incorporating Indigenous place-based relationality perspective in FS practice needs a stronger push emphasizing its well-being benefits for humans as well as natural environments. This perspective, along with custodial ethic of care, fosters a uniquely interconnected relationship with land/Country/place/nature, specifically instilling in children a place-based sense of identity. This allows children to connect with local Indigenous cultures, stories, knowledges, and perspectives. Integrating these into the FS adaptations of bush kinder, nature play, and outdoor kindergartens promotes culturally responsive pedagogies supporting reciprocal place-based relationships.

The synergies between Western and Indigenous perspectives enrich the FS experience in children. The Western focus on the person-environment relationship complements the Indigenous perspective of relationality with place/nature, influencing individual social and emotional well-being. This approach supports self-esteem, agency, identity formation, and a deepened sense of connection with the natural world. Graham’s (2014) custodial ethic of care concept resonates with this approach, emphasizing the significance of relationships for social and emotional well-being.

The FS’s adaptability to diverse cultural contexts contributes to community well-being and supports the dissemination of TEK. By catering to different socio-cultural groups, FS practitioners are encouraged to develop programs that emphasize the synergies between Western and local TEK, facilitating children’s development as well-rounded individuals with place-based identity and belonging.

Besides innovating FS practice further to suit local-specific contexts, there is also a need for building research evidence studying the perspectives of all stakeholders on the program efficacy with emphasis on embedding Indigenous worldviews. Focusing on exploring further the affordances along with place-based relationality in FS adaptations needs careful consideration and the involvement of local Indigenous communities for deepening the impact. This entails co-designing research projects that incorporate mixed methods and qualitative methods to evaluate the experience of FS along with the impact of embedding Indigenous place-based relationality concept in practice. Non-Indigenous researchers could collaborate with Indigenous community members to apply Indigenous research methodologies of Storywork and Yarning besides using ethnography and action research. Specific research questions should also address participants’ lived experience and the overall benefits for their social and emotional well-being. Further research examining FS program outcomes and impact in its local-specific variations should be carried out in different parts of the world to explore the sociocultural dimensions of FS adaptations, as well as to gain deeper insights into the synergies of affordances and place-based relationality.

## Conclusion

FS emerged as a robust child-centered experiential learning practice, offering a holistic approach to child development using natural environments. FS model’s focus on improving self-esteem and social skills supports children’s social, emotional, and physical well-being through immersive, nature-based activities. Going beyond play pedagogies, FS can benefit from the Western theory of affordances for strengthening person-environment relationship. When applied to cross-cultural contexts such as in European settler societies, FS practitioners can consider the incorporation of Indigenous perspective of place-based relationality for fostering reciprocal relationships between participants and the natural world. The affordances and place-based relationality perspectives enrich participants’ respectful interactions and relationships with nature, cultivating culturally responsive practices. The adaptability of FS to various cultural contexts embedding affordances together with local-specific Indigenous worldviews and TEK underscores its potential to enhance social and emotional well-being, promoting behavior, principles of ethics and values of caring for the place in its holistic sense. As this paper highlights, the synergies of affordances and place-based relationality hold transformative potential, extending their benefits beyond individual participants to families and communities, thereby nurturing more environmentally aware and emotionally resilient societies. Further research is needed to examine the impact of FS adaptations along with those synergies enhancing well-being outcomes. The authors have called for research focusing on co-designed studies involving local Indigenous communities and for the application of qualitative and mixed-methods study design evaluating FS program impact on individual and community well-being.

## Data availability statement

The original contributions presented in the study are included in the article/supplementary material, further inquiries can be directed to the corresponding author.

## Author contributions

VS-B: Conceptualization, Writing – original draft, Writing – review & editing. EB: Conceptualization, Writing – original draft, Writing – review & editing. RW: Conceptualization, Writing – original draft, Writing – review & editing. ML: Conceptualization, Writing – original draft, Writing – review & editing.

## References

[ref1] Alcock S. Ritchie J. (2018). Early childhood education in the outdoors in Aotearoa New Zealand. J. Outdoor Environ. Educ. 21, 77–88. doi: 10.1007/s42322-017-0009-y

[ref2] Barnes M. R. Donahue M. L. Keeler B. L. Shorb C. M. Mohtadi T. Z. Shelby L. J. (2019). Characterizing nature and participant experience in studies of nature exposure for positive mental health: an integrative review. Front. Psychol. 9:2617. doi: 10.3389/fpsyg.2018.02617, PMID: 30662418 PMC6329281

[ref3] Bentsen P. Stevenson M. P. Mygind E. Barfod K. S. (2018). “Udeskole: education outside the classroom in a Danish context” in The budding and blooming of outdoor education in diverse global contexts. eds. Huang M. T. Jade Ho Y. C. (Taiwan: National Academy for Educational Research), 81–114.

[ref4] Biedenweg K. Scott R. P. Scott T. A. (2017). How does engaging with nature relate to life satisfaction? Demonstrating the link between environment-specific social experiences and life satisfaction. J. Environ. Psychol. 50, 112–124. doi: 10.1016/j.jenvp.2017.02.002

[ref5] Boileau E. Y. S. Dabaja Z. F. (2020). Forest School practice in Canada: a survey study. J. Outdoor Environ. Educ. 23, 225–240. doi: 10.1007/s42322-020-00057-4

[ref6] Brymer E. Crabtree J. King R. (2021). Exploring perceptions of how nature recreation benefits mental wellbeing: a qualitative enquiry. Ann. Leisure Res. 24, 394–413. doi: 10.1080/11745398.2020.1778494

[ref7] Brymer E. Davids K. (2014). Experiential learning as a constraint-led process: an ecological dynamics perspective. J. Adv. Educ. Out. Learn. 14, 103–117. doi: 10.1080/14729679.2013.789353

[ref8] Bunyaville Environmental Education Centre. (n.d.). Bush explorers play group. Available at: https://bunyavilleeec.eq.edu.au/ourcurriculum/PreSchool/Pages/Bush-explorers.aspx

[ref9] Christiansen A. Hannan S. Anderson K. Coxon L. Fargher D. (2018). Place-based nature kindergarten in Victoria, Australia: no tools, no toys, no art supplies. J. Outdoor Environ. Educ. 21, 61–75. doi: 10.1007/s42322-017-0001-6

[ref10] Coates J. K. Pimlott-Wilson H. (2019). Learning while playing: Children’s Forest School experiences in the UK. Br. Educ. Res. J. 45, 21–40. doi: 10.1002/berj.3491

[ref11] Cooley S. J. Robertson N. Jones C. R. Scordellis J.-A. (2021). “Walk to wellbeing” in community mental health: urban and green space walks provide transferable biopsychosocial benefits. Ecopsychology 13, 84–95. doi: 10.1089/eco.2020.0050

[ref12] Coventry P. A. Brown J. V. E. Pervin J. Brabyn S. Pateman R. Breedvelt J.. (2021). Nature-based outdoor activities for mental and physical health: systematic review and meta-analysis. SSM Popul. Health 16, 1–14. doi: 10.1016/j.ssmph.2021.100934, PMID: 34646931 PMC8498096

[ref13] Cumming F. Nash M. (2015). An Australian perspective of a Forest School: shaping a sense of place to support learning. J. Adv. Educ. Out. Learn. 15, 296–309. doi: 10.1080/14729679.2015.1010071

[ref14] Dabaja Z. F. (2022). The Forest School impact on children: reviewing two decades of research. Education 50, 640–653. doi: 10.1080/03004279.2021.1889013

[ref15] Gee G. Dudgeon P. Schultz C. Hart A. Kelly K. (2014). “Aboriginal and Torres Strait islander social and emotional wellbeing” in working together: aboriginal and Torres Strait islander mental health and wellbeing principles and practice. eds. Dudgeon P. Milroy H. Walker R. (Canberra, Australia: Commonwealth Government of Australia).

[ref16] Gelter H. (2000). Friluftsliv: the Scandinavian philosophy of outdoor life. Can. J. Environ. Educ. 5, 77–90,

[ref17] Gelter H. (2010). Friluftsliv as slow and peak experiences in the transmodern society. Norwegian Journal of Friluftsliv. Available at: http://urn.kb.se/resolve?urn=urn:nbn:se:ltu:diva-9185.

[ref18] Gibson J. J. (1979). The ecological approach to visual perception. Boston: Houghton Mifflin.

[ref19] Graham M. (2008). Some thoughts about the philosophical underpinnings of aboriginal worldviews. Aust. Humanit. Rev. 45, 181–194,

[ref20] Graham M. (2013). The concept of ethics in Australian aboriginal systems of thought. Custodial Navigator. Available at: http://www.indigenoussovereigntyaustralia.com.au/wpcontent/uploads/2013/06/CustodialNavigator.pdf (Accessed July 12, 2023).

[ref21] Graham M. (2014). Aboriginal notions of relationality and positionalism: a reply to weber. Global Discourse 4, 17–22. doi: 10.1080/23269995.2014.895931

[ref22] Graham M. (2023). The law of obligation, aboriginal ethics: Australia becoming, Australia dreaming. Parrhesia 37, 1–21,

[ref23] Gregis A. Ghisalberti C. Sciascia S. Sottile F. Peano C. (2021). Community garden initiatives addressing health and well-being outcomes: a systematic review of infodemiology aspects, outcomes, and target populations. Int. J. Environ. Res. Public Health 18:4. doi: 10.3390/ijerph18041943, PMID: 33671320 PMC7922762

[ref24] Harris F. (2021). Developing a relationship with nature and place: the potential role of Forest School. Environ. Educ. Res. 27, 1214–1228. doi: 10.1080/13504622.2021.1896679

[ref25] Harris F. (2023). Practitioners’ perspectives on children’s engagement in Forest School. Education 3-13, 1–10. doi: 10.1080/03004279.2023.2183081

[ref26] Heft H. (1988). Affordances of children’s environments: a functional approach to environmental description. Environ. Psychol. Res. 5, 29–37,

[ref27] Jimenez M. P. Deville N. V. Elliott E. G. Schiff J. E. Wilt G. E. Hart J. E.. (2021). Associations between nature exposure and health: a review of the evidence. Int. J. Environ. Res. Public Health 18:4790. doi: 10.3390/ijerph18094790, PMID: 33946197 PMC8125471

[ref28] Kelly J. White J. E. Dekker M. Donald J. Hart K. McKay F.. (2013). The Ngahere project: teaching and learning possibilities in nature settings. New Zealand: Wilf Malcolm Institute of Educational Research Hamilton.

[ref29] Kimmerer R. (2013). Braiding sweetgrass: Indigenous wisdom, scientific knowledge, and the teachings of plants. Minneapolis, USA: Milkweed Editions.

[ref30] Knight S. (2012). Forest School for all. London, UK: SAGE Publications.

[ref31] Knight S. (2018). Translating Forest School: a response to leather. J. Outdoor Environ. Educ. 21, 19–23. doi: 10.1007/s42322-017-0010-5

[ref32] Lawton E. Brymer E. Clough P. Denovan A. (2017). The relationship between the physical activity environment, nature relatedness, anxiety, and the psychological well-being benefits of regular exercisers. Front. Psychol. 8, 1–11. doi: 10.3389/fpsyg.2017.01058, PMID: 28694788 PMC5483473

[ref33] Lee-Hammond L. Jackson-Barrett E. (2017). “Indigenous perspectives on outdoor learning environments: on country learning” in Outdoor learning environments. ed. Little H. (London, UK: Routledge).

[ref34] MacEachren Z. (2013). The Canadian Forest School movement. Learn. Land. 7, 219–233. doi: 10.36510/learnland.v7i1.639

[ref35] MacEachren Z. (2018). First nation pedagogical emphasis on imitation and making the stuff of life: Canadian lessons for indigenizing Forest schools. J. Outdoor Environ. Educ. 21, 89–102. doi: 10.1007/s42322-017-0003-4

[ref36] McCartan C. Davidson G. Bradley L. Greer K. Knifton L. Mulholland A.. (2023). “Lifts your spirits, lifts your mind”: a co-produced mixed-methods exploration of the benefits of green and blue spaces for mental wellbeing. Health Expect. Int. J. Pub. Particip. Health Care Health Policy 26, 1679–1691. doi: 10.1111/hex.13773, PMID: 37128668 PMC10349228

[ref37] O'Brien L. (2009). Learning outdoors: the Forest School approach. Education 37, 45–60. doi: 10.1080/03004270802291798

[ref39] Roberts A. Hinds J. Camic P. M. (2020). Nature activities and wellbeing in children and young people: a systematic literature review. J. Adv. Educ. Out. Learn. 20, 298–318. doi: 10.1080/14729679.2019.1660195

[ref40] Roberts J. D. Rodkey L. R. Ray R. Knight B. Saelens B. E. (2017). Electronic media time and sedentary behaviors in children: findings from the built environment and active play study in the Washington DC area. Prev. Med. Rep. 6, 149–156. doi: 10.1016/j.pmedr.2017.02.021 28316911 PMC5350570

[ref41] Sharma-Brymer V. Brymer E. Gray T. Davids K. (2018). Affordances guiding Forest School practice: the application of the ecological dynamics approach. J. Outdoor Environ. Educ. 21, 103–115. doi: 10.1007/s42322-017-0004-3

[ref42] Speldewinde C. Campbell C. (2023). Bush Kinders: building young children’s relationships with the environment. Aust. J. Environ. Educ. 40, 7–21. doi: 10.1017/aee.2023.36

[ref43] Thornton S. Graham M. Burgh G. (2021). Place-based philosophical education: reconstructing ‘place’, reconstructing ethics. Child. Phil. 17, 01–29. doi: 10.12957/childphilo.2021.54696

[ref44] Watene K. (2016). Valuing nature: Māori philosophy and the capability approach. Oxf. Dev. Stud. 44, 287–296. doi: 10.1080/13600818.2015.1124077

[ref45] White M. P. Alcock I. Grellier J. Wheeler B. W. Hartig T. Warber S. L.. (2019). Spending at least 120 minutes a week in nature is associated with good health and wellbeing. Sci. Rep. 9, 7730–7711. doi: 10.1038/s41598-019-44097-3, PMID: 31197192 PMC6565732

[ref46] Woods C. Davids K. (2021). “You look at an ocean; I see the rips, hear the waves, and feel the currents”: dwelling and the growth of enskiled inhabitant knowledge. Ecol. Psychol. 33, 279–296. doi: 10.1080/10407413.2021.1965481

